# Optimal resource allocation model for COVID-19: a systematic review and meta-analysis

**DOI:** 10.1186/s12879-024-09007-7

**Published:** 2024-02-14

**Authors:** Yu-Yuan Wang, Wei-Wen Zhang, Ze-xi Lu, Jia-lin Sun, Ming-xia Jing

**Affiliations:** 1 Department of Preventive Medicine, School of Medicine, Shihezi University, Shihezi, 832003 PR China; 2 Key Laboratory for Prevention and Control of Emerging Infectious Diseases and Public Health Security, The Xinjiang Production and Construction Corps, Urumqi, China; 3 Department of Nutrition and Food Hygiene School of Public Health Tongji Medical College, Huazhong University of Science and Technology, Wuhan, China

**Keywords:** COVID-19, Resource allocation, Efficiency, Model, Meta-analysis

## Abstract

**Background:**

A lack of health resources is a common problem after the outbreak of infectious diseases, and resource optimization is an important means to solve the lack of prevention and control capacity caused by resource constraints. This study systematically evaluated the similarities and differences in the application of coronavirus disease (COVID-19) resource allocation models and analyzed the effects of different optimal resource allocations on epidemic control.

**Methods:**

A systematic literature search was conducted of CNKI, WanFang, VIP, CBD, PubMed, Web of Science, Scopus and Embase for articles published from January 1, 2019, through November 23, 2023. Two reviewers independently evaluated the quality of the included studies, extracted and cross-checked the data. Moreover, publication bias and sensitivity analysis were evaluated.

**Results:**

A total of 22 articles were included for systematic review; in the application of optimal allocation models, 59.09% of the studies used propagation dynamics models to simulate the allocation of various resources, and some scholars also used mathematical optimization functions (36.36%) and machine learning algorithms (31.82%) to solve the problem of resource allocation; the results of the systematic review show that differential equation modeling was more considered when testing resources optimization, the optimization function or machine learning algorithm were mostly used to optimize the bed resources; the meta-analysis results showed that the epidemic trend was obviously effectively controlled through the optimal allocation of resources, and the average control efficiency was 0.38(95%CI 0.25–0.51); Subgroup analysis revealed that the average control efficiency from high to low was health specialists 0.48(95%CI 0.37–0.59), vaccines 0.47(95%CI 0.11–0.82), testing 0.38(95%CI 0.19–0.57), personal protective equipment (PPE) 0.38(95%CI 0.06–0.70), beds 0.34(95%CI 0.14–0.53), medicines and equipment for treatment 0.32(95%CI 0.12–0.51); Funnel plots and Egger’s test showed no publication bias, and sensitivity analysis suggested robust results.

**Conclusion:**

When the data are insufficient and the simulation time is short, the researchers mostly use the constructor for research; When the data are relatively sufficient and the simulation time is long, researchers choose differential equations or machine learning algorithms for research. In addition, our study showed that control efficiency is an important indicator to evaluate the effectiveness of epidemic prevention and control. Through the optimization of medical staff and vaccine allocation, greater prevention and control effects can be achieved.

**Supplementary Information:**

The online version contains supplementary material available at 10.1186/s12879-024-09007-7.

## Introduction

The coronavirus disease (COVID-19) pandemic has had a profound impact on the development of the global economy and social life [1]. The epidemic has had an enormous impact on the global medical system [2]. The mortality rate of COVID-19 patients with weakened immunity is as high as 41.7%, and the mortality rate will be even worse if medical resources are insufficient [3]. In the United States, a lack of vaccine resources that prevented older adults from receiving booster doses would have resulted in US $6.7 million in direct health care costs and 3.7 quality-adjusted life-years lost over 180 days [4]. Similarly, in Brazil, one of the low- and middle-income countries with severe COVID-19 infection, patients could not receive timely treatment due to insufficient ICU beds, resulting in a mortality rate as high as 34.42% [5]. Therefore, the shortage of medical resources is an important obstacle to the prevention and control of infectious diseases.

Studies have shown that resource optimization can effectively avoid infection caused by resource constraints [6]. In previous studies on resource optimization, through retrospective cohort analysis, researchers collected the characteristics of COVID-19 infection, summarized historical experience, and provided references for resource optimization for possible future situations [7, 8]. Some scholars use some management methods, such as 6S and PDCA, to optimize the work system or process to achieve the purpose of rational allocation of resources [9]. These methods can solve the problem of resource shortages in the short term and with a small scope, and there is a time lag. However, emergent infectious diseases are characterized by a wide range of diseases and a long duration. In contrast, modeling to solve the resource optimization problem has certain advantages, which can quickly simulate the effect of resource optimization in various situations and is not limited by time and region.

The model used in resource optimization is also controversial. Seyed Ali Rakhshan et al. [10] suggested that machine learning methods are more accurate than transmission dynamics models for long-term predictions. However, the results from a study in Korea showed that transmission dynamics were more accurate than machine learning models [11]. Alaleh Azhir et al. also showed differences in the prediction effect of next-day mortality using three machine learning models [12]. The current systematic reviews mainly focus on the infection characteristics of susceptible populations [13, 14] and rarely consider the optimal allocation of resources. However, it is not clear how much effect different types of resource optimization can achieve. Therefore, this study aimed to sort out and analyze the related research on SARS-CoV-2 optimization models and evaluate the quality of research articles in this field. We systematically sorted out the application status and existing problems of the optimization model in COVID-19 resource allocation, and provided experience for formulating resource allocation plans for public health emergencies in the future.

## Materials and methods

### Search strategy

We searched the CNKI, WanFang, VIP, CBD, PubMed, Web of Science, Scopus and Embase databases to collect different types of SARS-CoV-2 resource allocation models. The search time limit was from January 1, 2019 to June 1, 2023.We used a combination of subject words and free words for retrieval. The search terms included: COVID-19 pneumonia, COVID-19, model, resource allocation, resource optimization, optimal control, epidemic control, etc. (Supplementary Table 1). The review protocol was registered in PROSPERO (CRD42023458855).

### Literature screening

Two reviewers independently screened the studies, extracted and cross-checked the data. If there were disagreements, they were resolved by discussion or consultation with a third author. Studies were included if they (1) were related to the SARS-CoV-2 resource allocation scheme; (2) used at least one allocation model; or (3) involved optimization simulation or data simulation. The strategies that only considered the optimization of epidemic control or strategy without specific resource allocation were excluded.

### Data extraction and bias assessment

The following data were extracted independently by two reviewers: general study information (authors, year of publication, country, study design, modeling methodology, outcomes of achieving objectives and resource optimization, etc.).

All the studies included in this paper were model studies. The ISPOR-SMDM task force, as a model-centered evaluation tool, not only includes the evaluation of the model itself, but also includes the evaluation of the statement of the problem, modeling purpose, and data type, which can well evaluate the quality of model studies [15].Tadele Girum et al. [16] also used the ISPOR-SMDM task force to evaluate the quality of modeling studies. Two reviewers screened items from the ISPOR-SMDM task force and assessed the quality of each study independently, including research problem description, problem transformation into modeling structure, parameter settings, resource types described, sensitivity analysis, and more (Supplementary Table 2). If the content of the included articles met the evaluation items, the evaluation was “yes”; otherwise, the evaluation was “no”, and the quality of the article was finally judged by the frequency of “yes or no”.

### Statistic analysis

The average control efficiency (ACE) formula was used to obtain the comprehensive index reflecting the optimization effect [17]. The formula is as follows:

$$Average\ \textrm{Control}\ Efficiency=\frac{\sum_{k=0}^n\left[\left(\frac{\left|{C}_{k1}-{O}_{k1}\right|}{C_{k1}}\right)+\left(\frac{\left|{C}_{k2}-{O}_{k2}\right|}{C_{k2}}\right)+\cdots \left(\frac{\left|{C}_n-{O}_n\right|}{C_n}\right)\right]}{n}.$$

In this formulation, C is the control value of the unoptimized resource allocation. O is the control value of the optimized resource allocation.

Funnel plot analysis of publication bias was performed by Review Manager 5.3 software, and sensitivity analysis was performed by STATA 17.0 software. *P* < 0.05 was considered statistically significant [18, 19].

## Results

**Fig. 1:**
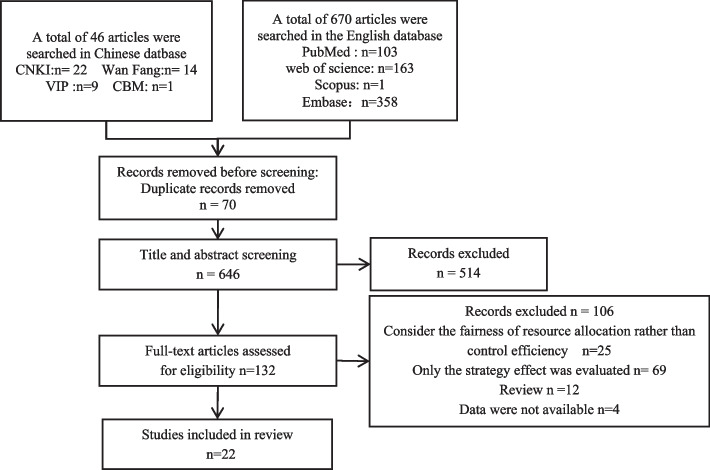
PRISMA (Preferred Reporting Items for Systematic Reviews and Meta-Analyses) flow diagram for the studies included in the current meta-analysis

A total of 716 relevant articles were obtained. After rechecking and reading titles and abstracts, only 132 articles were screened for full text, and 22 article were finally included in the systematic review (Fig. 1).

Among the 22 articles on resource allocation, 13 articles constructed differential equation models to achieve resource allocation. It is considered that a single optimal allocation model may have some limitations in allocating resources. Therefore, 4 articles utilized a combination of two or more methods for resource allocation (Table 1).

**Table 1:** Summary of optimal resource allocation models for COVID-19

Field	Frequency	Proportion of total (%)	References
**Total number of articles reviewed**	22	100	[20–41]
**Region of focus**			
Africa	3	13.64%	[20–22]
North America	6	27.27%	[22, 23, 25, 30, 35, 37]
Asia	7	31.82%	[24, 25, 31, 34, 36, 38, 39]
Europe	6	27.27%	[20, 25, 26, 28, 32, 34]
South America	1	4.55%	[25]
Numerical simulation	4	18.18%	[27, 29, 33, 40]
**Number of resources**			
1	18	81.82%	[20–24, 26, 28, 29, 31–36, 38–41]
2	1	4.55%	[37]
3 or more	3	13.64%	[25, 27, 30]
**Types of resources**			
Tests	4	18.18%	[21, 26–28]
Vaccines	6	27.27%	[29, 31, 32, 36, 40, 41]
Beds	8	36.36%	[20, 25, 27, 30, 34, 37–39]
Apparatus and Instruments	3	13.64%	[23, 30, 37]
PPE (Personal Protective Equipment)	2	9.09%	[25, 33]
Health specialists	3	13.64%	[24, 25, 27]
Therapeutic Drug	3	13.64%	[23, 30, 37]
Lockdown	1	4.55%	[22]
**Decision variables**			
confirmed cases/Infection rate	14	63.64%	[20–23, 25, 27–31, 33, 36, 40, 41]
Deaths/the mortality rate	9	40.91%	[25, 26, 30, 32–34, 38, 40, 41]
the recovery rate	4	18.18%	[24, 30, 35, 39]
cost-effectiveness	4	18.18%	[27, 37, 40, 41]
the duration of the epidemic	1	4.55%	[21]
**Method for modeling**			
differential equation (SEIR)	13	59.09%	[21, 22, 25–29, 31–34, 39–41]
optimization function	8	36.36%	[20, 22, 24, 32, 35, 36, 38]
machine learning algorithm	7	31.82%	[22, 23, 25, 26, 30, 37, 38]

### Results of article quality evaluation

Most of the studies met the conditions for the application of the model, but 59.09% of the articles did not perform sensitivity analysis, which is an important indicator to determine the accuracy of the model. In addition, 54.55% of the studies did not provide parameter settings, initial value tables or related accessory materials, which could not be verified by other scholars through the original data (Supplementary Table 3 and Fig. 1).

### Applicability analysis of the resource allocation optimization model

There are several prerequisites in model construction and resource allocation (Table 2). First, it is necessary to consider whether the data can provide the parameters of the model. In the large amount of available information and data, it is difficult to quantify the dynamic variables. Among the 22 studies included in this study, 4 studies used numerical simulation to study the problem of resource allocation. Therefore, appropriate methods could be selected to solve the problem of resource allocation. Second, suitable models were selected for the allocation of different resource types. From the included literature, differential equation modeling was more considered more when testing resource optimization. The optimization function or machine learning algorithm was mostly used to optimize the bed resources. Finally, the appropriate model is selected for implementation according to the optimization goal formulated in the study. The optimization goal can be divided into two situations: first, the maximization goal, such as resource coverage and efficiency, and second, the minimization goal, such as the prevention and control cost and demand.

**Table 2:** Optimal resource allocation models for COVID-19 included in review

Study identifier /year of publication	Geographic focus	Types of resources	Optimization technique	Optimization goal(s)
Evans et al. (2023)	Madagascar	Testing capacity	Epidemic Model (SEIR model)	Maximize testing efficiency
Xia,Zeyu et al. (2023)	numerical simulation	Testing Capacity; Beds; doctors and nurses	SEIR model	The cost-optimal solution for effective epidemic control
Jin Zhu et al. (2023)	England	Vaccines	the multi-period two-dose vaccine allocation model	Minimize lower vaccine supply levels and minimize the daily number of deaths
Barnieh L et al. (2023)	US	Patient-treating drugs; Beds	decision tree model、a Markov model	Minimize treatment (hospitalization、quality-adjusted life year) costs
Kai Zong et al. (2022)	US	Lockdown resource allocation	MARAAC structure、the advantage function、SEAIRD model	Minimize the economic loss while keeping the number of individuals
Khan A A et al. (2022)	Pakistan	Vaccines	a compartment epidemic model、 the compartmental-based COVID-19 vaccine model	Maximize vaccination
Schmidt et al. (2021)	Munich	Beds	A Planning Model for Intrahospital Resource Allocation	Maximize hospitalization rate
Apornak et al. (2021)	Iran	nurses	the linear programming technique	Maximize nurse service timer period
Libin et al. (2021)	Belgian	Testing capacity	extend the STRIDE model	Maximize testing efficiency
Daniel Kim et al. (2021)	numerical simulation	Vaccines	Extended SIR-D model	Maximize vaccine efficacy and reach
Jeongmin Kim et al. (2021)	Korea	ICU Beds	Multivariate logistic regression (LR) and XGBoost	Maximize hospitalization rate
Worby et al. (2020)	numerical simulation	masks	the “resource allocation”model、 the “supply & demand” model (SEIR model)	Maximize mask use
Michail et al. (2020)	Switzerland	Testing capacity	a sequential optimization algorithm、SEIrIuR epidemiological model	Minimize prediction uncertainty, Maximize information gain of unreported infections
Arunmozhi et al. (2022)	10 countries	Ventilators; PPE; ICU Beds; Health specialists	the Probability Queueing Theory (PQT) and K-Mean clustering Machine Learning (ML)	Increasing Capacity
Majid et al. (2023)	Iran	Vaccines	a two-stages model with uncertainty demand	Minimize the total cost of meeting demand、the maximum coverage index
Lin Wang et al. (2022)	US	ICU Beds; Ventilators; treatments for symptoms	a novel Lasso Logistic Regression model based on feature-based time series data	Reducing the mortality rate of hospitalized COVID-19 patients
Bing Xue et al. (2022)	US	ECMO	Multi-horizon machine learning prediction models	Maximize ECMO use
Ying-Qi Zeng et al. (2022)	4 countries	Beds	COVID-19 patient admission model	Maximize hospitalization rate
Mehrotra et al. (2020)	US	Ventilators	a multi-period planning model	Minimize ventilators’ shortage
Zhou D et al.(2022)	numerical simulation	Vaccines	a transmission dynamic-model	Minimizing the size of infection
Sean Shao et al.(2022)	Singapore	Beds	Beds resource planning model	Increasing beds Capacity
Krishna P. R et al.(2021)	South Africa	Vaccines	Micro simulation model	Minimize treatment costs

### Meta-analysis of control efficiency in resource allocation optimization

**Fig. 2:**
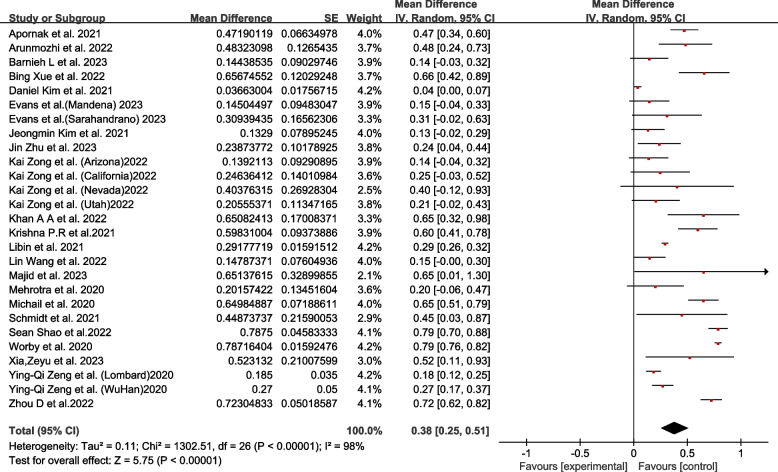
Forest plot of the average control efficiency in resource optimization

A total of 22 articles were included to calculate the control efficiency. According to the combined effect analysis, the epidemic trend was obviously effectively controlled through the optimal allocation of resources, and the average control efficiency was 0.38 (95% CI 0.25–0.51; I^2^ = 98%, *P* < 0.01) (Fig. 2).

According to the type of resources allocated, it was divided into different subgroups. The average control efficiency from high to low was health specialists 0.48 (95% CI 0.37–0.59; I^2^ = 0%, *P* < 0.01), vaccines 0.47 (95% CI 0.11–0.82; I^2^ = 98%, *P* = 0.01), tests 0.38 (95% CI 0.19–0.57; I^2^ = 86%, *P* < 0.01), personal protective equipment (PPE) 0.38 (95% CI 0.06–0.70; I^2^ = 94%, *P* = 0.02), beds 0.34 (95% CI 0.14–0.53; I^2^ = 94%, *P* < 0.01), medicines and equipment for treatment 0.32 (95% CI 0.12–0.51; I^2^ = 78%, *P* < 0.01)proved that comprehensive

 (Fig. 3).

**Fig. 3:**
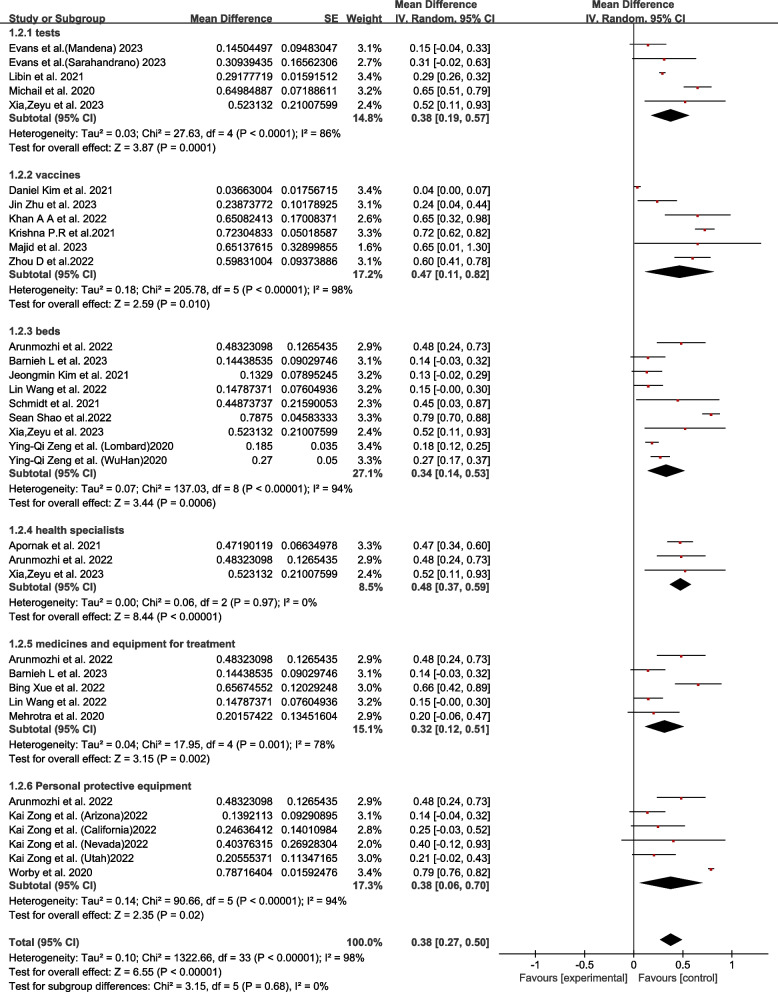
Results of subgroup analysis

### Publication bias and sensitivity analysis

The funnel plot results were basically symmetric, suggesting a possible minor publication bias (Supplementary Fig. 2). In addition, we used Egger’s test to verify the results and found that there was no publication bias (*P* = 0.7119).

Sensitivity analysis showed robust results (Supplementary Fig. 3).

## Discussion

In resource-constrained areas, resource optimization for infectious disease prevention and control urgently needs to be addressed. Most researchers use modeling studies to solve resource optimization problems [42, 43]. However, due to the differences in the models constructed, the resources allocated and the outcome indicators selected, the effects that can be achieved after resource optimization are also different. The systematic review and meta-analysis of the current model studies on optimizing resource allocation highlighted the importance of optimization objectives, optimization tools, and optimization resource types to evaluate and improve the efficiency of COVID-19 control. By systematically combing the articles applying the resource optimization model, we put forward the key issues that should be considered in modeling research and discussed the effect of optimizing resources.

Currently, to compare the accuracy of models, the same data are mostly used to use different models for simulation [44]. However, due to the inconsistent application conditions of different models, the data types used have a great impact on them. Meanwhile, the quality evaluation results of our included articles showed that the models rarely provided parameter settings and sources of values. The reliability of models will directly affect the formulation of optimization strategies, therefore, researchers need to understand the applicability of the model [45]. We divided the models used in the included literature into three types of resource allocation models according to their basic principles. Among them, the transmission dynamics model had at least three differential equations, which needed many parameters. Generally, the number of infections was estimated by iterative and summation methods. A study from Cameroon used a transmission dynamics model with 9 differential equations and 25 parameters to assess the impact of an intervention on transmission [46]. The optimization function requires fewer restrictions, which is suitable for less information. It is mainly constructed according to the purpose of the author, and there is no fixed framework, such as limit formula and expectation formula. A study in Brazil used three parameters to construct a model to simulate different control strategies and their cost-benefit analyses [47]. The machine learning algorithm mainly considers the impact of time on infectious diseases and is preferred when the prediction time is longer. This was also confirmed by the results of Dairi A et al. [48].

Control efficiency is an important indicator to evaluate the effectiveness of epidemic prevention and control. Xinru Wan et al. [49] used the control efficiency to reflect the transmission of SARS-CoV-2 under different temperatures and humidities. A Korean study explored the attitudes and work stress of school nurses to improve the efficiency of school infection control [50]. Resource optimization played an enormous role in the effect of epidemic control, especially when resources were limited. The results of our meta-analysis showed that the epidemic trend of COVID-19 had been effectively controlled through the optimal allocation of resources, with an average control efficiency of 0.38 (95% CI 0.25–0.51). Lin Xie et al. [51] explored the relationship between medical resources and the mortality of COVID-19 patients in Hubei Province, and found that the number of hospital beds, the number of beds in the health care system, and the number of medical staff in a unit with confirmed cases all had a significant negative impact. This is consistent with the results of this study. Resource optimization plays a role in controlling other infectious diseases. Studies by some scholars have shown that through resource optimization, AIDS, influenza A and other infectious diseases can also be rapidly controlled [52, 53]. In addition, the subgroup analysis of this study showed that the optimization of human resources, vaccine resources, testing resources and personal protection resources could achieve greater prevention and control effects, which may be related to the importance of various resources in prevention and control, but also indirectly reflects the difficulty of improving different prevention and control resources. A study in Morocco showed that the local government made various efforts to control the outbreak but lacked human resources, especially qualified human resources in intensive care and resuscitation [54]. There are differences in epidemic prevention strategies in different countries, the amount of resources is not consistent, and there are different resistances in the optimization process. However, the optimization of any type of medical resource can reduce the number of infections. Xia Wang et al. [55] also proved that comprehensive improvement of resource allocation ability can effectively reduce the infection rate.

### Study limitations

Our study included 22 articles on resource optimization models, but there are still some limitations. First, due to language limitations, only Chinese and English studies were included in the study, and there may be selection bias in the selection of included studies. Second, there is a certain heterogeneity in the included literature, which is not only related to the subgroup analysis of resource type, but also related to the modeling method selected by the researchers, data time period, outcome indicators and other factors. Further research can be carried out in subgroups. Third, the meta-analysis method of the single group rate was used in this paper, which makes it difficult to control for heterogeneity, and it needs to be further confirmed by other methods. Finally, only the resource type of the included literature was classified and analyzed, but there were many other resources that were not further analyzed.

## Conclusions

When the data are insufficient and the simulation time is short, the researchers mostly use the constructor for research; when the data are relatively sufficient and the simulation time is long, researchers choose differential equations or machine learning algorithms for research. In addition, our study showed that control efficiency is an important indicator to evaluate the effectiveness of epidemic prevention and control. Through the optimization of medical staff and vaccine allocation, greater prevention and control effects can be achieved. However, subsequent research should focus on improving the quality of research, improving the accuracy of the model, and establishing a simulation model that is closer to the real world.

### Supplementary Information

**Additional file 1.**

## Data Availability

The datasets used and/or analyzed during the current study are available from the corresponding author upon reasonable request.
