# Supplementary material for: Optimal resource allocation model for COVID-19: a systematic review and meta-analysis
Source: BMC Infect Dis. 2024 Feb 14;24:200. doi: 10.1186/s12879-024-09007-7 (PMC10865525; doi:10.1186/s12879-024-09007-7)
Supplement: Supplementary file 1 — Additional file 1. [file 12879_2024_9007_MOESM1_ESM.docx]

**SUPPLEMENTARY MATERIAL**

| **Supplementary Table 1 Search strategy** | | |
| --- | --- | --- |
| Database | Specific strategy（20190101-20230601January 1, 2019 - June 1, 2023） | Number of studies |
| CNKI | SU =(COVID-19+SARS-CoV-2) AND SU =(model) AND SU =(resource allocation+resource optimization + optimum control + Epidemic control) | 22 |
| WanFang | (Title or keywords:(COVID-19) or Title or keywords:(SARS-CoV-2)) and Title or keywords:(model) and (Title or keywords:(resource allocation) or Title or keywords:(resource optimization) or Title or keywords:(optimum control) or Title or keywords:(Epidemic control)) | 14 |
| VIP | (((Title or keywords=COVID-19 OR Title or keywords=SARS-CoV-2) AND Title or keywords=model) AND (((Title or keywords=resource allocation OR Title or keywords=resource optimization) OR Title or keywords=optimum control) OR Title or keywords=Epidemic control)) | 9 |
| CBM | ("COVID-19"[Keywords] AND "model"[Title]) AND ("resource allocation"[Keywords] OR "resource optimization"[Keywords] OR "resource optimization"[Keywords] OR "resource optimization"[Keywords]) | 1 |
| PubMed | ((((((((((COVID-19[Title]) OR (SARS-CoV-2[Title])) OR (2019 Novel Coronavirus Disease[Title])) OR (2019-nCoV Disease[Title])) OR (COVID-19 Virus Infection[Title])) OR (Coronavirus Disease 2019[Title])) AND ((model[Title])) OR (modeling[Title]))) AND ((resource allocation[Title]) OR (resource optimization[Title])) OR (optimum control[Title])) OR (Epidemic control[Title])) | 112 |
| Web of Science | ((((((TI=(COVID-19)) OR TI=(SARS-CoV-2)) OR TI=(2019 Novel Coronavirus Disease)) OR TI=(2019-nCoV Disease)) OR TI=(COVID-19 Virus Infection)) OR TI=(Coronavirus Disease 2019)) AND (TS=(model) OR TS=(modeling)) AND ((((TI=(resource allocation)) OR TI=(resource optimization)) OR TI=(optimum control)) OR TI=(Epidemic control)) | 199 |
| Scopus | (TITLE-ABS-KEY(COVID-19 OR SARS-CoV-2 OR 2019 Novel Coronavirus Disease OR 2019-nCoV Disease OR COVID-19 Virus Infection OR Coronavirus Disease 2019)) AND (TITLE-ABS-KEY(model OR modeling)) AND (TITLE-ABS-KEY(resource allocation OR resource optimization OR optimum control OR Epidemic control)) | 1 |
| Embase | ('covid 19':ti,ab,kw OR 'sars cov 2':ti,ab,kw OR '2019 novel coronavirus disease':ti,ab,kw OR '2019-ncov disease':ti,ab,kw OR 'covid-19 virus infection':ti,ab,kw OR 'coronavirus disease 2019':ti,ab,kw) AND (model:ti,ab,kw OR modeling:ti,ab,kw) AND ('resource allocation':ti,ab,kw OR 'resource optimization':ti,ab,kw OR 'optimum control':ti,ab,kw OR 'epidemic control':ti,ab,kw) | 358 |

| **Supplementary Table 2 Selected best practices for mathematical modeling from the joint ISPOR-SMDM task force** | |
| --- | --- |
| Recommendation number | Description |
| II-2 | A clear, written statement of the decision problem, modeling objective, and scope of the model should be developed. This should include: the spectrum of disease considered, perspective of the analysis, target population, alternative interventions, health and other outcomes, and time horizon |
| II-6 | An explicit process (expert consultations, influence diagrams, concept mapping, or similar method) should be used to convert the conceptualization of the problem into an appropriate model structure to ensure that the model reflects current theory of disease or the process being modeled. |
| IV-2 | Constrained resource models should consider health-related outcomes, and not focus solely on measures of throughput. |
| V-2 | The appropriate type of dynamic transmission model should be used for the analysis in question, based in part on the complexity of the interactions as well as the size of the population of interest and the role of chance effects. This model could be deterministic or stochastic, and population or individual-based. Justification for the model structure should be given. |
| V-7 | If using a differential equations model, provide the model equations. Tabulate all initial values and parameters, including the mixing matrix and supply details of the type of mixing considered. |
| VI-8 | When there is very little information on a parameter, analysts should adopt a conservative approach such that the absence of evidence is reflected in a very broad range of possible estimates. On no account should parameters be excluded from a sensitivity analysis on the grounds that ‘there is not enough information from which to estimate uncertainty |
| VII-4 | Models should be subjected to rigorous verification. The verification methods should be described in the non-technical documentation of the model. The pertinent results of verification should be made available on request |
| VII-5 | Modelers should search for previously published modeling analyses of the same or similar problems and discuss insights gained from similarities and differences in results. |

**Supplementary Table 3 Article quality evaluation**

| **Study identifier (year of publication)** | **Statement of decision problem** | **Statement of modeling objective** | **the conceptualization of the problem into an appropriate model structure** | **Health and other outcomes described** | **Differential equations for model included** | **Data time description** | **Parameters and initial values tabulated** | **Parameters and initial values labeled and described** | **Constrained resource described** | **Types of resources described** | **model framework described** | **Assessment of uncertainty performed** | **Non-technical documentation included** | **Linkage with previous modeling analyses** | **Sensitivity analysis** | |
| --- | --- | --- | --- | --- | --- | --- | --- | --- | --- | --- | --- | --- | --- | --- | --- | --- |
|  |  |  |  |  |  |  |  |  |  |  |  |  |  |  | **Yes or No** | **Type** |
| Evans et al. (2023) | Yes | Yes | Yes | Yes | Yes | Yes | No | No | Yes | Yes | Yes | Yes | Yes | Yes | Yes | Scenario |
| Xia,Zeyu et al. (2023) | Yes | Yes | Yes | Yes | Yes | No | No | No | Yes | Yes | Yes | No | No | Yes | No | NA |
| Jin Zhu et al. (2023) | Yes | Yes | Yes | Yes | Yes | Yes | No | Yes | Yes | Yes | Yes | Yes | Yes | Yes | No | NA |
| Barnieh L et al. (2023) | Yes | Yes | Yes | No | NA† | Yes | Yes | Yes | Yes | Yes | Yes | Yes | No | Yes | No | NA |
| Kai Zong et al. (2022) | Yes | Yes | Yes | Yes | Yes | Yes | Yes | Yes | Yes | Yes | Yes | Yes | No | Yes | Yes | Univariate |
| Khan A A et al. (2022) | Yes | Yes | Yes | Yes | Yes | Yes | Yes | Yes | Yes | Yes | Yes | Yes | No | Yes | Yes | Scenario |
| Schmidt et al. (2021) | Yes | Yes | Yes | Yes | NA† | Yes | No | Yes | Yes | Yes | Yes | Yes | Yes | Yes | No | NA |
| Apornak et al. (2021) | Yes | Yes | Yes | Yes | NA† | Yes | Yes | Yes | Yes | Yes | Yes | Yes | No | No | No | NA |
| Libin et al. (2021) | Yes | Yes | Yes | Yes | Yes | No | Yes | Yes | Yes | Yes | Yes | Yes | Yes | Yes | Yes | Scenario |
| Daniel Kim et al. (2021) | Yes | Yes | Yes | Yes | Yes | No | No | Yes | Yes | Yes | Yes | Yes | Yes | Yes | No | NA |
| Jeongmin Kim et al. (2021) | Yes | Yes | Yes | Yes | NA† | Yes | Yes | Yes | Yes | Yes | Yes | Yes | Yes | Yes | No | NA |
| Worby et al. (2020) | Yes | Yes | Yes | Yes | Yes | No | No | Yes | Yes | Yes | Yes | Yes | Yes | Yes | No | NA |
| Michail et al. (2020) | Yes | Yes | Yes | Yes | Yes | Yes | No | Yes | Yes | Yes | Yes | Yes | No | Yes | No | NA |
| Arunmozhi et al. (2022) | Yes | Yes | Yes | Yes | Yes | Yes | No | Yes | Yes | Yes | Yes | Yes | No | Yes | Yes | Scenario |
| Majid et al. (2023) | Yes | Yes | Yes | Yes | NA† | Yes | No | Yes | Yes | Yes | Yes | Yes | No | Yes | Yes | Scenario |
| Lin Wang et al. (2022) | Yes | Yes | Yes | Yes | NA† | Yes | Yes | No | Yes | Yes | Yes | No | No | Yes | Yes | Scenario |
| Bing Xue et al. (2022) | Yes | Yes | Yes | Yes | NA† | Yes | Yes | Yes | Yes | Yes | Yes | Yes | Yes | Yes | Yes | Scenario |
| Ying-Qi Zeng et al. (2020) | Yes | Yes | Yes | Yes | Yes | Yes | No | Yes | Yes | Yes | Yes | Yes | Yes | Yes | No | NA |
| Mehrotra et al. (2020) | Yes | Yes | Yes | Yes | NA† | Yes | No | Yes | Yes | Yes | Yes | Yes | Yes | Yes | No | NA |
| Zhou D et al.(2022) | Yes | Yes | No | Yes | Yes | No | No | No | No | Yes | No | No | No | No | Yes | Scenario |
| Sean Shao et al.(2022) | Yes | Yes | Yes | No | Yes | Yes | Yes | Yes | Yes | Yes | Yes | Yes | Yes | Yes | Yes | Scenario |
| Krishna P.R et al.(2021) | Yes | Yes | Yes | Yes | NA† | No | Yes | Yes | Yes | Yes | Yes | Yes | Yes | Yes | Yes | Scenario |
| NA, not applicable  †Does not utilize a mathematical transmission model | | | | | | | | | | | | | | | | |

**Supplementary Figure 1 Results of quality assessment**


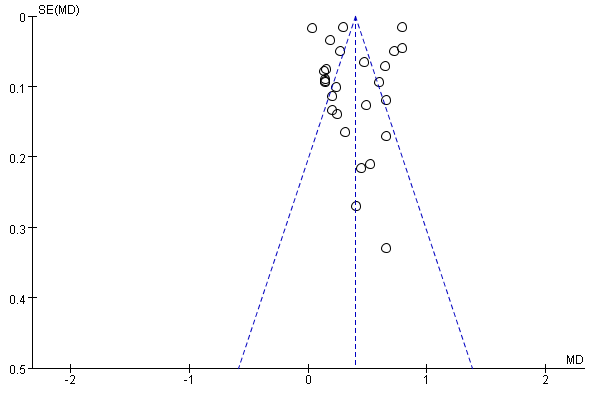


**Supplementary Figure 2 Publication of bias assessment results**

**Supplementary Figure 3 sensitivity analysis**
